# Fetal cerebral blood‐flow redistribution: analysis of Doppler reference charts and association of different thresholds with adverse perinatal outcome

**DOI:** 10.1002/uog.23615

**Published:** 2021-11-01

**Authors:** H. Wolf, T. Stampalija, C. C. Lees, the TRUFFLE Study Group, B. Arabin, A. Berger, E. Bergman, A. Bhide, C. M. Bilardo, A. C. Breeze, J. Brodszki, P. Calda, E. Cesari, I. Cetin, J. Derks, C. Ebbing, E. Ferrazzi, T. Frusca, W. Ganzevoort, S. J. Gordijn, W. Gyselaers, K. Hecher, P. Klaritsch, L. Krofta, P. Lindgren, S. M. Lobmaier, N. Marlow, G. M. Maruotti, F. Mecacci, K. Myklestad, R. Napolitano, F. Prefumo, L. Raio, J. Richter, R. K. Sande, J. Thornton, H. Valensise, G. H. A. Visser, L. Wee

**Affiliations:** ^1^ Department of Obstetrics and Gynecology, Amsterdam University Medical Center (Location AMC) University of Amsterdam Amsterdam The Netherlands; ^2^ Unit of Fetal Medicine and Prenatal Diagnosis Institute for Maternal and Child Health, IRCCS Burlo Garofolo Trieste Italy; ^3^ Department of Medicine, Surgery and Health Sciences University of Trieste Trieste Italy; ^4^ Department of Metabolism, Digestion and Reproduction, Imperial College London London UK; ^5^ Centre for Fetal Care, Queen Charlotte's and Chelsea Hospital, Imperial College NHS Trust London UK

**Keywords:** adverse outcome, brain sparing, cerebroplacental ratio, Doppler, fetal growth restriction, middle cerebral artery, percentile, reference chart, umbilicocerebral ratio

## Abstract

**Objectives:**

First, to compare published Doppler reference charts of the ratios of flow in the fetal middle cerebral and umbilical arteries (i.e. the cerebroplacental ratio (CPR) and umbilicocerebral ratio (UCR)). Second, to assess the association of thresholds of CPR and UCR based on these charts with short‐term composite adverse perinatal outcome in a cohort of pregnancies considered to be at risk of late preterm fetal growth restriction.

**Methods:**

Studies presenting reference charts for CPR or UCR were searched for in PubMed. Formulae for plotting the median and the 10^th^ percentile (for CPR) or the 90^th^ percentile (for UCR) against gestational age were extracted from the publication or calculated from the published tables. Data from a prospective European multicenter observational cohort study of singleton pregnancies at risk of fetal growth restriction at 32 + 0 to 36 + 6 weeks' gestation, in which fetal arterial Doppler measurements were collected longitudinally, were used to compare the different charts. Specifically, the association of UCR and CPR thresholds (CPR < 10^th^ percentile or UCR ≥ 90^th^ percentile and multiples of the median (MoM) values) with composite adverse perinatal outcome was analyzed. The association was also compared between chart‐based thresholds and absolute thresholds. Composite adverse perinatal outcome comprised both abnormal condition at birth and major neonatal morbidity.

**Results:**

Ten studies presenting reference charts for CPR or UCR were retrieved. There were large differences between the charts in the 10^th^ and 90^th^ percentile values of CPR and UCR, respectively, while median values were more similar. In the gestational‐age range of 28–36 weeks, there was no relationship between UCR or CPR and gestational age. From the prospective observational study, 856 pregnancies at risk of late‐onset preterm fetal growth restriction were included in the analysis. The association of abnormal UCR or CPR with composite adverse perinatal outcome was similar for percentile thresholds or MoM values, as calculated from the charts, and for absolute thresholds, both on univariable analysis and after adjustment for gestational age at measurement, estimated fetal weight MoM and pre‐eclampsia. The adjusted odds ratio for composite adverse perinatal outcome was 3.3 (95% CI, 1.7–6.4) for an absolute UCR threshold of ≥ 0.9 or an absolute CPR threshold of < 1.11 (corresponding to ≥ 1.75 MoM), and 1.6 (95% CI, 0.9–2.9) for an absolute UCR threshold of ≥ 0.7 to < 0.9 or an absolute CPR threshold of ≥ 1.11 to < 1.43 (corresponding to ≥ 1.25 to < 1.75 MoM).

**Conclusions:**

In the gestational‐age range of 32 to 36 weeks, adjustment of CPR or UCR for gestational age is not necessary when assessing the risk of adverse outcome in pregnancies at risk of fetal growth restriction. The adoption of absolute CPR or UCR thresholds, independent of reference charts, is feasible and makes clinical assessment simpler than if using percentiles or other gestational‐age normalized units. The high variability in percentile threshold values among the commonly used UCR and CPR reference charts hinders reliable diagnosis and clinical management of late preterm fetal growth restriction. © 2021 The Authors. Ultrasound in Obstetrics & Gynecology published by John Wiley & Sons Ltd on behalf of International Society of Ultrasound in Obstetrics and Gynecology.

**CONTRIBUTION**

**What are the novel findings of this work?**

Comparison of different cerebroplacental ratio (CPR) or umbilicocerebral ratio (UCR) reference charts demonstrated large differences in threshold values. In pregnancies at risk of late preterm fetal growth restriction (FGR) (32 + 0 to 36 + 6 weeks), absolute thresholds for CPR or UCR had a similar association with adverse short‐term perinatal outcome as did percentile thresholds or thresholds based on other gestational‐age normalized units.

**What are the clinical implications of this work?**

Absolute UCR or CPR thresholds can be used for clinical management of late preterm FGR. This makes fetal assessment simpler and does not require Doppler reference charts.

## INTRODUCTION

Doppler assessment of the fetal umbilical and cerebral circulations has become important in the diagnosis and management of fetal growth restriction (FGR)^1^, ^2^. Cerebral blood‐flow redistribution (otherwise known as ‘brain sparing’) indicates preferential fetal cardiac output redistribution toward the brain, heart and adrenal glands in the presence of hypoxemia^3^. It can be evaluated through the assessment of fetal middle cerebral artery (MCA) pulsatility index (PI), systolic/diastolic ratio or resistance index, or the ratio between MCA‐PI and umbilical artery (UA) PI, namely the so‐called cerebroplacental ratio (CPR)^4^ and umbilicocerebral ratio (UCR)^5^. Several methods for relating cerebral blood‐flow redistribution to gestational age have been proposed, such as computing percentiles, multiples of the median (MoM), *Z*‐scores or absolute values^6^. Calculation of the ratio of MCA‐PI to UA‐PI (i.e. CPR) has gained prominence, and CPR < 5^th^ percentile has been proposed to define cerebral blood‐flow redistribution^2^. This requires the use of Doppler reference charts with accurate and reproducible percentile thresholds.

Many studies have reported an association between cerebral blood‐flow redistribution and adverse short‐ and long‐term outcomes in FGR^7^, ^8^, ^9^, ^10^, ^11^, ^12^. However, the value of cerebral blood‐flow redistribution ratios in determining the timing of delivery is still not known^13^, ^14^, ^15^. This uncertainty may, at least in part, be due to the heterogeneity of reference charts for fetal arterial Doppler parameters^16^, some of which may be explained by methodological shortcomings. Ruiz‐Martinez *et al*.^17^ performed a simulation analysis in a cohort of small‐for‐gestational‐age fetuses using percentile thresholds from the 10 most frequently cited Doppler reference charts for UA‐PI, MCA‐PI and CPR. They concluded that the variation in percentile thresholds could result in large variation in clinical management. The objectives of the current study were: (1) to compare published Doppler reference charts of the ratios of MCA‐PI and UA‐PI (i.e. CPR and UCR); and (2) to assess the association of thresholds of these reference charts with short‐term adverse perinatal outcomes in a cohort of pregnancies considered to be at risk of late preterm FGR.

## METHODS

### Doppler reference chart selection and evaluation

Reference charts that described values for UA‐PI and MCA‐PI and their ratio (UCR or CPR), within the gestational‐age range of 20–40 weeks in a normal population, were selected from PubMed using the search string: ““*((“1970/01/01”[Date‐Publication*] *: “2020/07/10”[Date ‐ Publication])) AND ((((reference chart)[Title/Abstract] OR (reference value))[Title/Abstract] AND ((cerebro‐placental)[Title/Abstract] OR (cerebroplacental)[Title/Abstract] OR (umbilical cerebral artery)[Title/Abstract] OR (umbilical‐cerebral)[Title/Abstract] OR (umbilicocerebral))[Title/Abstract] AND (ratio))[Title/Abstract])*””. The aim was to identify studies that allowed the extraction of a formula using gestational age for the calculation of median values as well as the 10^th^ percentile values for CPR or the 90^th^ percentile values for UCR. If such formulae were not available, data from published tables specifying the median and 10^th^ or 90^th^ percentile values for each week of gestation were used to determine a gestational‐age**‐**dependent formula. The data were fitted in polynomial models of increasing order, and the model with the highest *R* ^2^ was selected. If only mean and SD were reported, then it was assumed that the data distribution was normal, and these were used for calculation of the 10^th^ or 90^th^ percentiles. If the 10^th^ or 90^th^ percentiles were not available in the publication but the 5^th^ or 95^th^ percentiles were provided, the 10^th^ or 90^th^ percentiles were calculated as (mean ± 1.282 × ((95^th^ percentile – 50^th^ percentile)/1.645)) for each week of gestation. Studies in which a mean or median value for each week of gestation was not available were excluded from further assessment. Data from all 10^th^ percentile (CPR), 90^th^ percentile (UCR) and median formulae were plotted against gestational age.

### Clinical assessment of Doppler reference charts

#### Study population

Data were collected during a prospective multicenter observational study conducted between 1^st^ April 2017 and 1^st^ July 2018 in 33 European perinatal centers with fetal medicine and specialized neonatal intensive care services. Observational data from this study have been described previously^12^. In brief, women were eligible if they had a singleton pregnancy at 32 + 0 to 36 + 6 weeks' gestation with a fetus considered to be at risk for growth restriction, defined as estimated fetal weight (EFW) or abdominal circumference (AC) < 10^th^ percentile, abnormal arterial Doppler or a fall in AC growth velocity of at least 40 percentile points from the 20‐week scan. The references for EFW, AC and Doppler parameters were based on local charts. Fetuses with absent end‐diastolic flow in the UA, an abnormal cardiotocogram, an immediate indication for delivery or a structural abnormality were not eligible. Pre‐eclampsia was defined as hypertension and proteinuria, or hypertension and clinical signs of pre‐eclampsia, at any time during pregnancy^18^.

#### Study endpoint

The primary outcome was a composite of abnormal condition at birth, major neonatal morbidity and mortality. Abnormal condition at birth was defined as at least one of the following: Apgar score < 7 at 5 min, UA pH < 7.0, umbilical vein pH < 7.1, need for resuscitation with intubation, chest compressions or medication, or stillbirth. Major neonatal morbidity was defined as at least one of the following: neurological abnormality (intracerebral hemorrhage Grade 3 or 4, periventricular leukomalacia Grade 2 or 3, encephalopathy or seizures necessitating antiepileptic drug treatment), cardiovascular abnormality (hypotensive treatment, ductus arteriosus treatment or disseminated coagulopathy), respiratory morbidity (respiratory support for more than 1 week, mechanical ventilation, meconium aspiration or persistent pulmonary hypertension) or sepsis (clinical sepsis with positive blood culture, necrotizing enterocolitis (Bell's Stage 2 or greater) or meningitis).

### Statistical analysis

Using all selected reference charts, the median and 10^th^ or 90^th^ percentile values were calculated, based on the gestational age at measurement. CPR measurements < 10^th^ percentile or UCR measurements ≥ 90^th^ percentile were coded as abnormal. Median values from reference charts for CPR were transformed to 1/UCR or *vice versa*. However, percentiles were not transformed because the distributions of UCR and CPR are skewed in an opposite direction and transformation may have caused bias. For each UCR or CPR measurement, MoM was calculated as: observed value/median value for gestational age.

The fetal arterial Doppler measurement obtained at inclusion was selected from each woman for assessment of the association between composite adverse perinatal outcome and the 10^th^ and 90^th^ percentile thresholds of CPR and UCR, respectively, based on the selected reference charts. True‐positive and false‐negative rates and odds ratios (OR) with 95% CIs were calculated. Furthermore, ORs with 95% CIs were calculated for the association of all threshold values with composite adverse perinatal outcome, with adjustment for gestational age at measurement, EFW MoM and pre‐eclampsia, using logistic regression analysis.

The predictive value of all models was estimated by plotting a receiver‐operating‐characteristics (ROC) curve and calculating the area under the curve (AUC). The coordinates of the curves were used to determine the sensitivity and specificity of the models for the prediction of composite adverse outcome. This analysis was repeated for absolute UCR and CPR threshold values to assess if MoM values, which were adjusted for gestational age, had a better predictive value than unadjusted UCR or CPR for the composite adverse perinatal outcome.

From the available reference chart data, we aimed to ascertain if a UCR or CPR threshold value that improves the prediction of composite adverse perinatal outcome could be selected.

### Ethical approval

This study was observational, and practice (monitoring, delivery and steroid administration) was based on existing local guidance. Data were recorded and anonymized after delivery outcomes had been ascertained. In six countries (19 centers), ethical approval was required and obtained, and participating women gave informed written consent. In the remaining five countries, this was not required.

## RESULTS

### Doppler reference chart selection

**Figure 1:**
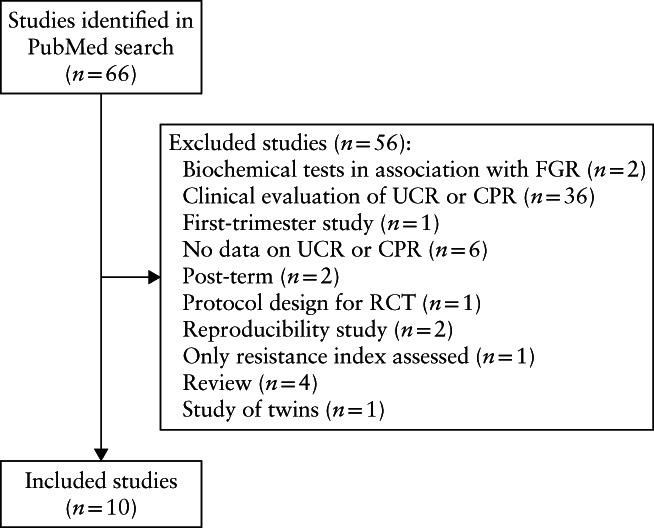
Flowchart showing selection of studies reporting reference charts of ratios of middle cerebral artery and umbilical artery pulsatility index (i.e. cerebroplacental ratio (CPR) or umbilicocerebral ratio (UCR)). FGR, fetal growth restriction; RCT, randomized controlled trial.

The PubMed search identified 66 publications. Figure 1 shows a flowchart of the selection process. Ten studies complied with the selection criteria and were included in the study^19^, ^20^, ^21^, ^22^, ^23^, ^24^, ^25^, ^26^, ^27^, ^28^. Table S1 summarizes the characteristics of the selected studies. Two studies recruited women prospectively^21^, ^24^; all other studies selected data from women who visited the antenatal clinic.

Three of the included studies reported reference formulae for the mean values of CPR or UCR^19^, ^20^, ^21^. Three studies contained a formula for the median^22^, ^23^, ^24^. For the four other studies, gestational‐age**‐**dependent formulae for the median were determined from the published table data of the median values^25^, ^26^, ^27^, ^28^.

Two studies reported a formula for percentiles^22^, ^24^. For two charts, the percentiles were calculated from a published formula for the mean or median and SD^21^, ^23^, while in two other studies, a formula for the mean was available, but the formula for SD had to be determined from the tables^19^, ^20^. For the remaining four charts, the SD was derived from table data for the percentile values^25^, ^26^, ^27^, ^28^. Formulae were determined from chart data using a polynomial model with an order (second or third) that achieved the highest *R* ^2^ value.

### Doppler reference chart evaluation

**Figure 2:**
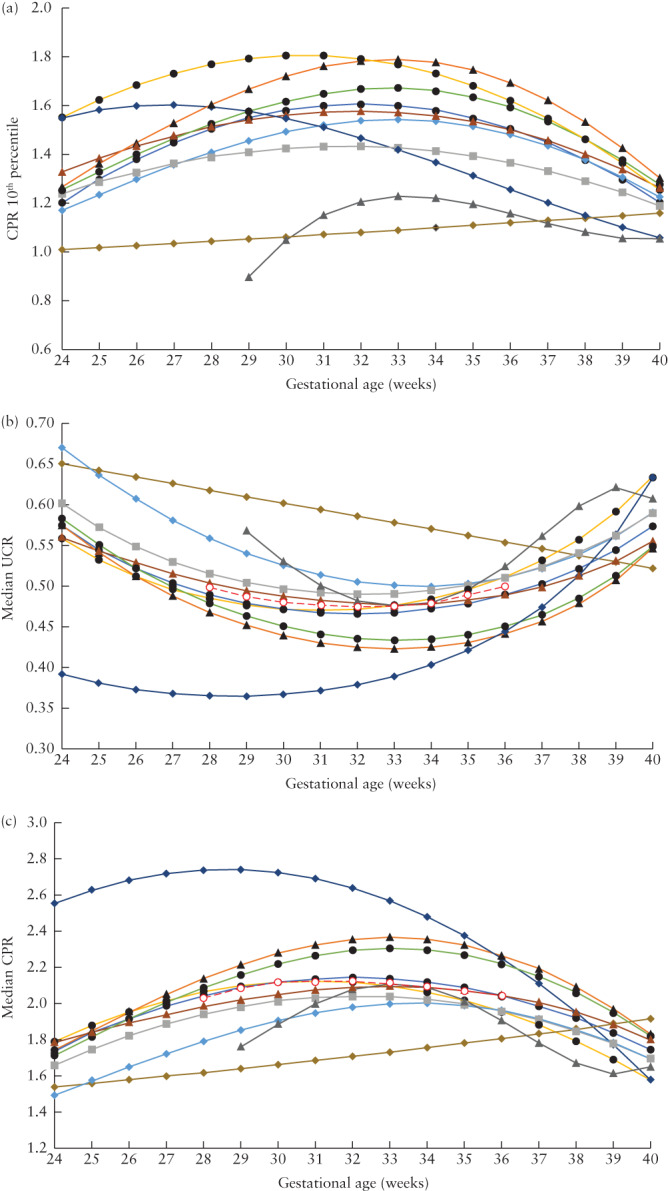
Cerebroplacental ratio (CPR) 10^th^ percentile values (a) and median umbilicocerebral ratio (UCR) (b) and CPR (c) values for different reference charts, according to gestational age. The UCR charts of Arduini and Rizzo^19^ were transformed to CPR (1/UCR) in (a) and (c); all other charts were for CPR and were transformed to UCR (1/CPR) in (b). Only first author of study is given: 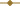 Arduini (1990)^19^; 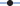, Baschat (2003)^20^; 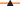, Ebbing (2007)^21^; 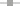, Morales‐Rosello (2015)^22^; 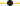, Srikumar (2017)^26^; 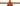, Flatley (2019)^25^; 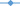, Ciobanu (2019)^23^; 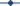, Dias (2019)^27^; 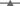, Zohav (2019)^28^; 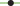, Acharya (2020)^24^; 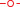, Median of all medians in (b) and (c).

For all charts, the 10^th^ percentile of CPR was plotted against gestational age (Figure 2a). Because all studies, except one^19^, reported CPR data, CPR was used for the plot, and UCR data from the study of Arduini and Rizzo^19^ were transformed to CPR (1/UCR). This was done for ease of presentation, although this transformation might have caused some bias. Variation in the percentile threshold values between the charts was large; in the most clinically relevant gestational‐age period of 28 to 36 weeks, the 10^th^ percentile value of CPR varied between approximately 0.9 and 1.8.

Figure 2b shows the median UCR values for all reference charts. There were two outliers: the charts of Dias *et al*.^27^ and of Arduini and Rizzo^19^. The chart of Dias *et al*.^27^ had far higher CPR values (or lower UCR values) in the earlier gestational‐age range of 24–30 weeks, as compared with the other charts. All charts had a polynomial profile, except for the chart of Arduini and Rizzo^19^, which had a linear profile and differed from the other charts in the gestational‐age window of 28–35 weeks. When the charts of Dias *et al*.^27^ and Arduini and Rizzo^19^ were excluded, the range between the median values of UCR in the remaining eight charts was less than 0.1. Figure 2c shows a plot of the median values of CPR. When the charts of Dias *et al*.^27^ and Arduini and Rizzo^19^ were excluded, the range between the median values of CPR was approximately 0.4, which was proportionally similar to the variation observed for UCR.

Assessment of the graph of UCR median values for the different charts (Figure 2b) showed that, in the gestational‐age range of 28 to 36 weeks, the profile of the median values was nearly linear and not related to gestational age. The pooled median of all reference charts in the range of 28 to 36 weeks had an ellipsoid pattern (Figure 2b). However, the difference between the highest UCR values, at 28 and 36 weeks, and the nadir at 33 weeks was only 0.025. For CPR, plotting the pooled median of all charts (Figure 2c) showed that the difference between the lowest values, at 28 and 36 weeks, and the peak at 31 weeks was 0.09. These differences were both roughly 5% of the average value of the respective parameter.

### Clinical assessment of Doppler reference charts

In the prospective study, complete delivery and outcome data were recorded for 873 patients at risk of late‐onset preterm FGR in the study database. Seventeen cases were excluded because of the presence of a major congenital abnormality, leaving 856 pregnancies for the final cohort analysis. Demographic, obstetric and fetal Doppler velocimetry characteristics of the included pregnancies are shown in Table 1 and neonatal outcome is given in Table 2.

**Table 1:** Demographic and obstetric characteristics of study population of 856 singleton pregnancies at risk for late‐onset preterm fetal growth restriction

Variable	Value
Maternal age (years)	31 (28–35)
Nulliparous	524 (61)
Body mass index (kg/m^2^)	22.5 (20.3–26.0)
Smoker	68 (8)
Diabetes (Type 1, 2 or gestational)	70 (8)
Chronic hypertension	19 (2)
At inclusion	
Gestational age (weeks)	34 (33–35)
Indication for inclusion*	
EFW or AC < 10^th^ percentile	792 (93)
AC growth velocity drop ≥ 40 percentile points	50 (6)
Doppler abnormality	98 (11)
EFW (g)	1894 (1624–2145)
EFW *Z*‐score	–1.52 (–2.0 to –1.1)
Umbilical artery PI	1.00 (0.86–1.14)
Middle cerebral artery PI	1.75 (1.51–2.01)
UCR	0.56 (0.47–0.69)
CPR	1.79 (1.45–2.14)
Before delivery	
Pre‐eclampsia or HELLP	79 (9)
Any hypertensive disorder of pregnancy	119 (14)
Corticosteroids for fetal lung maturation†	98 (11)
Total number of arterial Doppler measurements	2770
Number of arterial Doppler measurements per woman	3 (2–4)
Inclusion–delivery interval (days)	27 (14–38)
Delivery	
Planned CS for:	219 (26)
Fetal condition (CTG or Doppler)	155/219 (71)
Fetal growth/EFW	25/219 (11)
Maternal condition	39/219 (18)
Induction of labor for:	369 (43)
Fetal condition (CTG or Doppler)	112/369 (30)
Fetal growth/EFW	213/369 (58)
Maternal condition	44/369 (12)
Spontaneous onset of labor	268 (31)
CS after onset of labor	117/637 (18)

Data are presented as median (interquartile range), *n* (%) or *n*/*N* (%).

* Multiple indications possible.

† > 24 h before delivery.

AC, abdominal circumference; CPR, cerebroplacental ratio; CS, Cesarean section; CTG, cardiotocography; EFW, estimated fetal weight; PI, pulsatility index; UCR, umbilicocerebral ratio.

**Table 2:** Neonatal outcome of study population of 856 singleton pregnancies at risk for late‐onset preterm fetal growth restriction

Variable	Value
Gestational age at delivery (weeks)	38 (37–39)
Birth weight (g)	2478 (2140–2790)
Birth‐weight *Z*‐score	–1.7 (–2.3 to –1.1)
Birth weight < 10^th^ percentile	596 (70)
Male sex	372 (43)
Composite adverse outcome*	93 (11)
Abnormal condition at birth†	27 (3)
Fetal death	2 (0)
Umbilical artery pH < 7.0 or umbilical vein pH < 7.1‡	7 (1)
5‐min Apgar score < 7	15 (2)
Resuscitation with intubation or medication	10 (1)
Major neonatal morbidity†	77 (9)
Cerebral	7 (1)
Cardiovascular	7 (1)
Respiratory§	53 (6)
Infection	17 (2)
Neonatal death	0 (0)

Data are presented as median (interquartile range) or *n* (%).

* Defined as abnormal condition at birth or major neonatal morbidity or mortality.

† Multiple conditions possible.

‡ Data were missing for 17% of cases.

§ Of the 53 cases of respiratory morbidity, 39 (74%) had only some respiratory support of short duration in the first week postpartum.

Although UCR ≥ 90^th^ percentile or CPR < 10^th^ percentile was associated significantly with composite adverse outcome for all reference charts, the false‐negative rate was high (Table 3). The differences between the charts in the 90^th^ and 10^th^ percentiles caused variation in sensitivity (ranging from 15% in the study of Srikumar *et al*.^26^ to 28% in the study of Arduini and Rizzo^19^) and in specificity in the opposite direction (93% and 90%, respectively).

**Table 3:** Performance of umbilicocerebral ratio (UCR) ≥ 90^th^ percentile or cerebroplacental ratio (CPR) < 10^th^ percentile at inclusion, in the prediction of composite adverse perinatal outcome in 856 pregnancies at risk of late‐onset preterm fetal growth restriction, using different published reference charts

Study	Ratio assessed	TPR*	FPR	Specificity (%)†	Crude OR (95% CI)
Arduini (1990)^19^	UCR	18/64 (28)	75/792 (9)	90	3.7 (2.1–6.8)
Baschat (2003)^20^	CPR	51/270 (19)	42/586 (7)	93	3.0 (1.9–4.7)
Ebbing (2007)^21^	CPR	60/404 (15)	33/452 (7)	93	2.2 (1.4–3.5)
Morales‐Rosello (2015)^22^	CPR	39/184 (21)	54/672 (8)	92	3.1 (2.0–4.8)
Srikumar (2017)^26^	CPR	58/377 (15)	35/479 (7)	93	2.3 (1.5–3.6)
Ciobanu (2019)^23^	CPR	49/252 (19)	44/604 (7)	93	3.1 (2.0–4.8)
Dias (2019)^27^	CPR	39/161 (24)	54/695 (8)	92	3.8 (2.4–6.0)
Flatley (2019)^25^	CPR	50/263 (19)	43/593 (7)	93	3.0 (1.9–4.6)
Zohav (2019)^28^	CPR	22/84 (26)	71/772 (9)	91	3.5 (2.0–6.0)
Acharya (2020)^24^	CPR	53/339 (16)	40/517 (8)	92	2.2 (1.4–3.4)

Only first author's name is given for each study.

Data are given as *n*/*N* (%), unless indicated otherwise.

* True‐positive rate (TPR) is equal to sensitivity.

† Specificity is equal to 100 − false‐positive rate (FPR).

OR, odds ratio.

ORs for the association between composite adverse perinatal outcome and the reference chart thresholds (UCR ≥ 90^th^ percentile or CPR < 10^th^ percentile) were calculated using logistic regression analysis with adjustment for gestational age at measurement, EFW MoM and pre‐eclampsia (Table 4). The adjusted OR values varied from 1.7 to 2.7. However, the AUC of the ROC curve of the predicted probability for composite adverse perinatal outcome was similar for all charts (range, 0.71–0.73)^19^, ^20^, ^21^, ^22^, ^23^, ^24^, ^25^, ^26^, ^27^, ^28^. ORs for composite adverse perinatal outcome were also calculated for each reference chart using UCR MoM at inclusion, with adjustment for gestational age at measurement, EFW MoM at inclusion and pre‐eclampsia (Table 5). The adjusted ORs were similar for all charts (range, 1.5–1.7) and the AUCs of the ROC curve of the calculated probability of composite adverse perinatal outcome were the same (0.71 (95% CI, 0.66–0.77)). Repeating these calculations for CPR MoM produced similar results, with identical AUC values (Table 5).

**Table 4:** Association between composite adverse perinatal outcome and umbilicocerebral ratio (UCR) ≥ 90^th^ percentile or cerebroplacental ratio (CPR) < 10^th^ percentile at inclusion, adjusted for gestational age at measurement (GA), estimated fetal weight multiples of the median (EFW MoM) and pre‐eclampsia, in 856 pregnancies at risk for late‐onset preterm fetal growth restriction, using different published reference charts

Study	Ratio assessed	aOR (95% CI)	AUC (95% CI)
Arduini (1990)^19^	UCR	2.7 (1.4–5.1)	0.73 (0.67–0.78)
Baschat (2003)^20^	CPR	2.2 (1.4–3.5)	0.73 (0.67–0.78)
Ebbing (2007)^21^	CPR	1.7 (1.1–2.7)	0.71 (0.65–0.77)
Morales‐Rosello (2015)^22^	CPR	2.3 (1.4–3.8)	0.72 (0.66–0.78)
Srikumar (2017)^26^	CPR	1.7 (1.0–2.7)	0.71 (0.66–0.77)
Ciobanu (2019)^23^	CPR	2.2 (1.4–3.6)	0.72 (0.66–0.78)
Dias (2019)^27^	CPR	2.6 (1.6–4.3)	0.73 (0.67–0.78)
Flatley (2019)^25^	CPR	2.2 (1.4–3.5)	0.72 (0.66–0.78)
Zohav (2019)^28^	CPR	2.4 (1.3–4.4)	0.73 (0.67–0.78)
Acharya (2020)^24^	CPR	1.7 (1.0–2.6)	0.71 (0.65–0.77)
Adjustment parameters			
GA (in weeks)	—	0.8 (0.7–1.0)	—
EFW MoM	—	0.6 (0.4–0.7)	—
Pre‐eclampsia	—	2.1 (1.2–3.7)	—

Only first author's name is given for each study.

At a sensitivity of 70%, specificity was approximately 60% for all models.

aOR, adjusted odds ratio; AUC, area under receiver‐operating‐characteristics curve.

**Table 5:** Association between composite adverse perinatal outcome and umbilicocerebral ratio (UCR) or cerebroplacental ratio (CPR) multiples of the median (MoM) at inclusion (as a continuous variable), adjusted for gestational age at measurement (GA), estimated fetal weight (EFW) MoM and pre‐eclampsia, in 856 pregnancies at risk of late‐onset preterm fetal growth restriction, using different published reference charts

Study	Adjusted OR (95% CI)		AUC (95% CI)
	UCR MoM	CPR MoM	
Arduini (1990)^19^	1.74 (1.04–2.90)	0.45 (0.21–0.98)	0.71 (0.66–0.77)
Baschat (2003)^20^	1.58 (1.04–2.41)	0.38 (0.15–0.97)	0.71 (0.66–0.77)
Ebbing (2007)^21^	1.51 (1.03–2.22)	0.34 (0.12–0.96)	0.71 (0.66–0.77)
Morales‐Rosello (2015)^22^	1.62 (1.04–2.52)	0.39 (0.16–0.97)	0.71 (0.66–0.77)
Srikumar (2017)^26^	1.60 (1.04–2.47)	0.38 (0.15–0.97)	0.71 (0.66–0.77)
Ciobanu (2019)^23^	1.63 (1.04–2.56)	0.40 (0.16–0.97)	0.71 (0.66–0.77)
Dias (2019)^27^	1.46 (1.02–2.09)	0.33 (0.11–0.90)	0.71 (0.66–0.77)
Flatley (2019)^25^	1.59 (1.04–2.45)	0.38 (0.15–0.97)	0.71 (0.66–0.77)
Zohav (2019)^28^	1.60 (1.04–2.47)	0.38 (0.15–0.95)	0.71 (0.66–0.77)
Acharya (2020)^24^	1.53 (1.03–2.26)	0.35 (0.13–0.96)	0.71 (0.66–0.77)
Adjustment parameters			
GA (in weeks)	*0.86 (0.73–0.99)*		—
EFW MoM	*0.54 (0.41–0.71)*		—
Pre‐eclampsia	*2.27 (1.33–3.86)*		—

Only first author's name is given for each study.

At a sensitivity of 70%, specificity was approximately 60% for all models.

For assessment of CPR, the UCR charts of Arduini and Rizzo^19^ were transformed (1/UCR); all other charts were for CPR and were transformed (1/CPR) for assessment of UCR.

AUC, area under receiver‐operating‐characteristics curve; OR, odds ratio.

For the classification of UCR values, we used the pooled median of all charts for all gestational ages. UCR was categorized as < 1.25 MoM, ≥ 1.25 to < 1.75 MoM and ≥ 1.75 MoM, which corresponded to absolute UCR values of < 0.7, ≥ 0.7 to < 0.9 and ≥ 0.9, respectively, or absolute CPR values of ≥ 1.43, ≥ 1.11 to < 1.43 and < 1.11, respectively. These values were close to the average 75^th^ and 90^th^ percentiles calculated for UCR from the selected charts. Specifying these absolute UCR categories for gestational age at inclusion at 32–33 weeks, 34–35 weeks and 36 weeks showed that abnormal UCR ≥ 0.9 or CPR < 1.11 was associated significantly with composite adverse perinatal outcome in each gestational‐age category (Table 6). The absolute UCR/CPR thresholds, gestational age at measurement, EFW MoM and pre‐eclampsia were entered into a logistic regression model with composite adverse perinatal outcome as the dependent variable (Table 7). UCR ≥ 0.9 or CPR < 1.11 had an adjusted OR for composite adverse outcome of 3.3 (95% CI, 1.7–6.4). The AUC of the model was 0.73 (95% CI, 0.67–0.78), with a sensitivity of 70% and specificity of 64%.

**Table 6:** Rate of composite adverse perinatal outcome in 856 pregnancies at risk of late‐onset preterm fetal growth restriction, overall and according to umbilicocerebral ratio (UCR) and cerebroplacental ratio (CPR) absolute thresholds at inclusion and gestational age at measurement (GA)

GA	UCR/CPR value threshold			All
	< 0.7/≥ 1.43	≥ 0.7 to < 0.9/≥ 1.11 to < 1.43	≥ 0.9/< 1.11	
32–33 weeks	28/311 (9)	14/61 (23)*	12/29 (41)*	54/401 (13)
34–35 weeks	21/242 (9)	4/52 (8)	6/28 (21)*	31/322 (10)
36 weeks	5/105 (5)	2/22 (9)	1/6 (17)*	8/133 (6)
All (32–36 weeks)	54/658 (8)	20/135 (15)	19/63 (30)*	93/856 (11)

Data are given as *n*/*N* (%).

* Pearson χ‐square *P* < 0.05, compared with UCR < 0.7 or CPR ≥ 1.43.

**Table 7:** Association between composite adverse perinatal outcome and absolute thresholds of umbilicocerebral ratio (UCR) or cerebroplacental ratio (CPR) assessed at inclusion, gestational age at measurement (GA), estimated fetal weight multiples of the median (EFW MoM) and pre‐eclampsia, in 856 pregnancies at risk of late‐onset preterm fetal growth restriction

Parameter	aOR (95% CI)	*P*
UCR < 0.7/CPR ≥ 1.43	—	< 0.001
UCR ≥ 0.7 to < 0.9/CPR ≥ 1.11 to < 1.43	1.6 (0.9–2.9)	0.10
UCR ≥ 0.9/CPR < 1.11	3.3 (1.7–6.4)	< 0.001
GA (in weeks)	0.8 (0.7–0.9)	0.03
EFW MoM	0.6 (0.4–0.7)	< 0.001
Pre‐eclampsia	2.0 (1.2–3.5)	0.01

Area under receiver‐operating‐characteristics curve of the model was 0.73 (95% CI, 0.67–0.78), with sensitivity of 70% and specificity of 64%.

aOR, adjusted odds ratio.

## DISCUSSION

### Principal findings

Different reference charts for UCR and CPR are not directly comparable, but all demonstrate that the indices are not related to gestational age between 28 and 36 weeks. In prospectively collected observational data of women at risk for FGR at a gestational age of 32 + 0 to 36 + 6 weeks, percentiles, MoM values and absolute thresholds of UCR and CPR had a similar association with short‐term adverse perinatal outcome. There is, in our view, no reason to use percentile values of UCR or CPR for the clinical management of fetuses at risk of FGR, as the same information can be obtained more simply from absolute thresholds (at least in the gestational‐age window of 32 + 0 to 36 + 6 weeks, as assessed in our study), reducing the risk of error and variability.

We found large differences in reported percentile threshold values for CPR and UCR between different Doppler reference charts, which was similarly demonstrated in two previous studies^16^, ^17^. The most probable reason for these differences is an insufficient number of included cases for reliable determination of the limits of normality for each week of gestation, in addition to other methodological aspects^16^. As values close to normal are far more common than abnormal values, the variation in median values is much smaller than that for the percentile threshold values at the limits of normality, thus MoM values might be preferred over percentiles. However, because the median reference chart values have a nearly linear profile at 28 to 36 weeks, without a relationship with gestational age, adjustment for gestational age seems unnecessary, at least in fetuses in the gestational‐age window of 32 + 0 to 36 + 6 weeks, as assessed in the current study.

Based on the assessment of our observational data in pregnancies at 32 + 0 to 36 + 6 weeks, in relation to composite adverse perinatal outcome, an absolute UCR threshold of < 0.7 might be considered normal, ≥ 0.7 to < 0.9 as moderately abnormal and ≥ 0.9 as abnormal (with corresponding CPR thresholds of ≥ 1.43, ≥ 1.11 to < 1.43 and < 1.11, respectively). Two studies compared percentile CPR thresholds with absolute CPR thresholds for predicting adverse perinatal outcome in a cohort of pregnancies with FGR, and concluded that CPR < 1.08 (or UCR > 0.93) was as effective as the 5^th^ percentile of CPR^29^, ^30^. Indeed, a consensus meeting of experts in fetal medicine revealed that they would be willing to randomize pregnancies to delivery based on a UCR threshold of ≥ 0.8 from 34 weeks onwards, while for earlier gestational ages (< 34 weeks), a higher threshold of 1.0 was preferred^31^. This reflects the natural caution of clinicians, with the aim of delivering very preterm fetuses only when their condition is thought to be critical.

### Clinical implications

In the most clinically relevant gestational‐age period at which abnormalities of UA and MCA Doppler waveforms and their ratios might guide fetal management in FGR (28–36 weeks' gestation), the 10^th^ percentile value of the different charts for CPR varied from 0.9 to 1.8. These differences resulted in different sensitivities and ORs for composite adverse perinatal outcome and would also affect the proportion of FGR cases that are diagnosed, given that CPR is a criterion used in the diagnosis of this condition^2^. Our findings suggest that absolute UCR or CPR threshold values, rather than percentile thresholds or thresholds based on other gestational‐age normalized units, might be more practical for clinical use, with the added advantage that, as a simple ratio that is independent of gestational age, Doppler reference charts are not required.

### Research implications

The overall performance of ratios of UA‐PI and MCA‐PI in predicting composite adverse perinatal outcome in late preterm FGR from 32 + 0 to 36 + 6 weeks was poor, with a specificity of approximately 60% at a sensitivity of 70%, which does not make a strong case for the utility of these ratios for triggering delivery in a clinical setting. This inference must be placed in context; as in many similar studies, the results that we describe are influenced by clinical management. The major confounder for the interpretation of these results is obstetric intervention, namely delivery of the baby, which was aimed at diminishing the risk of adverse infant outcome. It is not possible to control for the effect of this intervention. It is also not possible to determine whether and how knowledge of the ratios of UA‐PI and MCA‐PI was used in making the decision for delivery. Furthermore, longer‐term outcome data in infancy were not available for this and other studies. Only a randomized controlled interventional trial based on an index of cerebral blood‐flow redistribution and with long‐term follow‐up may provide a definitive answer to the question of whether delivery based on cerebral blood‐flow redistribution in late preterm FGR is beneficial.

### Strengths and limitations

The main strength of this study is that UCR or CPR Doppler reference charts and absolute thresholds of the ratios were tested in a large, prospectively recruited cohort of fetuses at risk of late preterm FGR. However, it should be acknowledged that an inclusion criterion of the present cohort, derived from the TRUFFLE‐2 observational study^12^, was gestational age between 32 + 0 and 36 + 6 weeks, thus the results of this analysis can be applied only to this gestational‐age window. Furthermore, it should be taken into account that our study population had a high risk for adverse perinatal outcome. Application of Doppler thresholds for the prediction of adverse perinatal outcome in a low‐risk population will result in lower sensitivity and specificity. The findings of two Cochrane reviews support this; one showed an improvement in perinatal outcome associated with routine fetal Doppler assessment in a high‐risk population, while the other did not observe a benefit in low‐risk pregnant women^32^, ^33^. Lastly, as is common in all observational studies, the association between diagnostic criteria and outcome is influenced by clinical management.

There has been some debate in relation to whether CPR^34^, ^35^ or UCR^36^, ^37^ is preferred for describing the degree of cerebral redistribution. Our preference for UCR derives from the analysis of the early FGR TRUFFLE cohort^38^, in which UCR and MCA *Z*‐score, but not CPR, were associated with long‐term outcome. Moreover, most ratios used in medicine show progressively greater differentiation of values in the abnormal (not normal) range, which is the case for UCR but not CPR; this is true for the soluble fms‐like tyrosine kinase‐1/placental growth factor ratio for risk assessment of pre‐eclampsia, protein/creatinine ratio in the diagnosis of pre‐eclampsia and ventilation/perfusion ratio for ventilation–perfusion mismatch.

### Conclusions

The findings of this study confirm the presence of large differences between fetal Doppler reference charts for indices describing the relationship between UA and MCA impedance, particularly in the extreme ranges (< 10^th^ percentile or ≥ 90^th^ percentile), which might have a major clinical impact, particularly when used for the diagnosis and management of FGR. However, we observed that, in the gestational‐age window of 32 + 0 to 36 + 6 weeks, adjustment of UCR or CPR for gestational age had no advantage in the prediction of adverse outcome, as compared with absolute threshold values. This means that adoption of absolute UCR or CPR thresholds independent of Doppler reference charts is feasible, with these thresholds being simpler to use in clinical practice.

## TRUFFLE‐2 GROUP AND CONTRIBUTING AUTHORS

B. Arabin, Department of Obstetrics Charite, HumboldtUniversity Berlin and Clara Angela Foundation, Berlin,Germany

A. Berger, Department of Obstetrics and Gynecology,Medical University of Innsbruck, Innsbruck, Austria

E. Bergman, Department of Women's and Children'sHealth, Uppsala University, Uppsala, Sweden

A. Bhide, Fetal Medicine Unit, St George's UniversityHospitals NHS Foundation Trust, London, UK; Molecular & Clinical Sciences Research Institute, St George'sUniversity of London, London, UK

C. M. Bilardo, Department of Obstetrics and Gynecology, Amsterdam University Medical Centers, University of Amsterdam, Location VUMC, Amsterdam,The Netherlands

A. C. Breeze, Fetal Medicine Unit, Leeds GeneralInfirmary, Leeds Teaching Hospitals NHS Trust, Leeds,UK

J. Brodszki, Department of Pediatric Surgery and Neonatology, Lund University, Skane University Hospital,Lund, Sweden

P. Calda, Department of Obstetrics and Gynaecology,General University Hospital and First Faculty ofMedicine, Charles University, Prague, Czech Republic

E. Cesari, Department of Obstetrics and Gynecology,Vittore Buzzi Children's Hospital, University of Milan,Milan, Italy

I. Cetin, Department of Obstetrics and Gynecology,Vittore Buzzi Children's Hospital, University of Milan,Milan, Italy

J. Derks, Department of Perinatal Medicine, Universityof Utrecht, Utrecht, The Netherlands

C. Ebbing, Department of Obstetrics and Gynecology,Haukeland University Hospital, Bergen, Norway

E. Ferrazzi, Department of Obstetrics and Gynecology,Fondazione IRCCS Ca' Granda Ospedale MaggiorePoliclinico and Department of Clinical Sciences andCommunity Health, Università degli Studi di Milano,Milan, Italy

T. Frusca, Department of Obstetrics and Gynecology,University of Parma, Parma, Italy

W. Ganzevoort, Department of Obstetrics and Gynecology, Amsterdam University Medical Centers, location AMC, Amsterdam, The Netherlands

S. J. Gordijn, Department of Obstetrics and Gynaecology, University Medical Center Groningen, University of Groningen, Groningen, The Netherlands

W. Gyselaers, Faculty of Medicine and Life Sciences,Hasselt University, Agoralaan, Diepenbeek, Belgium;Department of Obstetrics & Gynaecology, ZiekenhuisOost‐Limburg, Genk and Department Physiology,Hasselt University, Diepenbeek, Belgium

K. Hecher, Department of Obstetrics and FetalMedicine, University Medical Centre Hamburg‐Eppendorf, Hamburg, Germany

P. Klaritsch, Department of Obstetrics and Gynecology,Medical University of Graz, Graz, Austria

L. Krofta, Institute for the Care of Mother and Child,Prague, Czech Republic; Third Medical Faculty, CharlesUniversity, Prague, Czech Republic

P. Lindgren, Center for Fetal Medicine, KarolinskaUniversity Hospital, Stockholm, Sweden

S. M. Lobmaier, Department of Obstetrics and Gynecology, Klinikum Rechts Der Isar, Technical Universityof Munich, Munich, Germany

N. Marlow, UCL Elizabeth Garrett Anderson Institutefor Women's Health, University College London,London, UK

G. M. Maruotti, Department of Neurosciences,Reproductive and Dentistry Sciences, University ofNaples ‘Federico II’, Naples, Italy

F. Mecacci, Department of Health Sciences, Universityof Florence, Obstetrics and Gynecology, CareggiUniversity Hospital, Florence, Italy

K. Myklestad, St Olav's Hospital, Trondheim, Norway

R. Napolitano, UCL Elizabeth Garrett AndersonInstitute for Women's Health, University CollegeLondon, London, UK; Fetal Medicine Unit, UniversityCollege London Hospitals NHS Foundation Trust,London, UK

F. Prefumo, Department of Obstetrics and Gynecology,ASST Spedali Civili di Brescia and University of Brescia,Brescia, Italy

L. Raio, Department of Obstetrics & Gynecology,University Hospital of Bern, Bern, Switzerland

J. Richter, Department of Gynecology and Obstetrics,UZ Leuven and Department of Regeneration andDevelopment, KU Leuven, Leuven, Belgium

R. K. Sande, Department of Obstetrics and Gynecology,Stavanger University Hospital, Stavanger and Department of Clinical Science, University of Bergen, Bergen,Norway

J. Thornton, School of Clinical Sciences, University ofNottingham, Division of Obstetrics and Gynaecology,Maternity Department, City Hospital, Nottingham, UK

H. Valensise, Department of Surgery, Division ofObstetrics and Gynecology, Tor Vergata, University,Policlinico Casilino Hospital, Rome, Italy

G. H. A. Visser, Department of Perinatal Medicine,University of Utrecht, Utrecht, The Netherlands

L. Wee, The Princess Alexandra Hospital NHS Trust,Harlow, UK

## Supporting information


**Appendix S1** TRUFFLE‐2 collaborating authors

**Table S1** Characteristics of included studies presenting Doppler reference charts

## Data Availability

The data that support the findings of this study are available from the corresponding author upon reasonable request.
