# Supplementary material for: Fetal cerebral blood‐flow redistribution: analysis of Doppler reference charts and association of different thresholds with adverse perinatal outcome
Source: Ultrasound Obstet Gynecol. 2021 Nov 1;58(5):705–15. doi: 10.1002/uog.23615 (PMC8597586; doi:10.1002/uog.23615)
Supplement: Supplementary file 1 — Appendix S1 TRUFFLE‐2 collaborating authors [file UOG-58-705-s001.docx]

**Appendix S1** TRUFFLE-2 collaborating authors

C. Brezinka, Department of Obstetrics and Gynecology, Medical University of Innsbruck, Innsbruck, Austria;

D. Casagrandi, University College London Hospitals NHS Foundation Trust, London, UK;

A. Cerny, Department of Obstetrics and Gynaecology, General University Hospital and First Faculty of Medicine, Charles University, Prague, Czech Republic;

A. Dall’Asta, Department of Obstetrics and Gynecology, University of Parma, Parma, Italy;

R. Devlieger, Department of Gynecology and Obstetrics, UZ Leuven, Leuven and Department of Regeneration and Development, KU Leuven, Leuven, Belgium;

J. Duvekot, Erasmus Centre Rotterdam, Rotterdam, The Netherlands;

T. M. Eggebo, St Olav’s Hospital, Trondheim, Norway;

I. Fantasia, Unit of Fetal Medicine and Prenatal Diagnosis, Institute for Maternal and Child Health, IRCCS Burlo Garofolo, Trieste, Italy;

F. Ferrari, Obstetrics & Gynecology, Policlinico University Hospital of Modena, Modena, Italy;

N. Fratelli, Department of Obstetrics and Gynecology, ASST Spedali Civili di Brescia and University of Brescia, Brescia, Italy;

T. Ghi, Department of Obstetrics and Gynecology, University of Parma, Parma, Italy;

O. Graupner, Department of Obstetrics and Gynecology, Klinikum Rechts Der Isar, Technical University of Munich, Munich, Germany;

P. Greimel, Department of Obstetrics and Gynecology, Medical University of Graz, Graz, Austria;

C. Hofstaetter, Department of Obstetrics & Gynecology, University Hospital of Bern, Bern, Switzerland;

D. Lo Presti, Department of Surgery, Division of Obstetrics and Gynaecology, Tor Vergata University, Policlinico Casilino Hospital, Rome, Italy;

M. Georg, Helsinki University Central Hospital, Helsinki, Finland;

A. Vietheer, Department of Obstetrics and Gynecology, Haukeland University Hospital, Bergen, Norway;

F. Macsali, Department of Obstetrics and Gynecology, Haukeland University Hospital, Bergen, Norway;

K. Marsal, Department of Obstetrics and Gynecology, Lund University, Sk°ane University Hospital, Lund, Sweden;

P. Martinelli, Department of Neurosciences, Reproductive and Dentistry Sciences, University of Naples ‘Federico II’, Naples, Italy; B. Mylrea-Foley, Imperial College London, London, UK;

E. Mullins, Imperial College London, London, UK;

E. Ostermayer, Department of Obstetrics and Gynecology, Klinikum Rechts Der Isar, Technical University of Munich, Munich, Germany;

A. Papageorghiou, Fetal Medicine Unit, St George’s University Hospitals NHS Foundation Trust and Molecular & Clinical Sciences Research Institute, St George’s, University of London, London, UK; R. Peasley, Fetal Medicine Unit, University College London Hospitals NHS Foundation Trust, London, UK;

A. Ramoni, Department of Obstetrics and Gynecology, Medical University of Innsbruck, Innsbruck, Austria;

L. Sarno, Department of Neurosciences, Reproductive and Dentistry Sciences, University of Naples ‘Federico II’, Naples, Italy;

L. Seikku, Helsinki University Central Hospital, Helsinki, Finland; S. Simeone, Department of Health Sciences, University of Florence, Obstetrics and Gynecology, Careggi University Hospital, Florence, Italy;

B. Thilaganathan, Fetal Medicine Unit, St George’s University Hospitals NHS Foundation Trust and Molecular & Clinical Sciences Research Institute, St George’s, University of London, London, UK;

G. Tiralongo, Department of Surgery, Division of Obstetrics and Gynaecology, Tor Vergata University, Policlinico Casilino Hospital, Rome, Italy;

A. Valcamonico, Department of Obstetrics and Gynecology, ASST Spedali Civili di Brescia and University of Brescia, Brescia, Italy;

C. Van Holsbeke, Department of Obstetrics & Gynaecology, Ziekenhuis Oost-Limburg, Genk, Belgium.
