# Supplementary material for: Fetal cerebral blood‐flow redistribution: analysis of Doppler reference charts and association of different thresholds with adverse perinatal outcome
Source: Ultrasound Obstet Gynecol. 2021 Nov 1;58(5):705–15. doi: 10.1002/uog.23615 (PMC8597586; doi:10.1002/uog.23615)
Supplement: Supplementary file 2 — Table S1 Characteristics of included studies presenting Doppler reference charts [file UOG-58-705-s002.docx]

**Table S1** Characteristics of included studies presenting Doppler reference charts

| Author | CPR or UCR | Years | Type study | N  Women | N measure-ments | Abnormal  pregnancies excluded | GA measurements (weeks) | Recruitment | Model published for | Table content | Table data | Median calculation | 10^th^ / 90^th^ percentile calculation |
| --- | --- | --- | --- | --- | --- | --- | --- | --- | --- | --- | --- | --- | --- |
| Arduini^19^ | UCR | ? | Crossectional | 1556 | 1556 | Yes | 20-42 | ANC | mean only | p5-mean-p95 | Modeled | published algorithm of mean | Published algorithm for mean, SD calculated from p50-p95 |
| Basschat^20^ | CPR | ? | Crossectional | 306 | 326 | Yes | 20-40 | ANC | mean only | mean, SD | Observed | published algorithm of mean | Published algorithm for mean, SD calculated from table |
| Ebbing^21^ | CPR | ? | Longitudinal | 161 | 550 | Low risk at inclusion, later pathology not excluded | 19-41 | prospective | mean + SD | 2.5 to p97.5 | Modeled | published algorithm of mean | Published algorithm for mean and SD |
| Morales^22^ | CPR | ? | Crossectional | 2323 | 2323 | Not excluded | 19-41 | ANC | p3 to p97 | p3 to p97 (including median) | Modeled | published algorithm ov median | Published algorithm for percentile |
| Srikumar^26^ | CPR | 2013-2015 | Longitudinal | 200 | 773 | Yes | 18-40 | ANC | None | p5, quartiles, p95, mean + SD | Observed | calculated from table median | Calculated from mean + SD in table |
| Flatley^25^ | CPR | 2010-2017 | Crossectional | 4464 | 4464 | Yes | 18-41 | ANC | None | p0.4 to 99.6 (including median) | Modeled | calculated fom table median | Calculated from table percentile |
| Ciobanu^23^ | CPR | 2011-2014 | Crossectional | 72387 | 72387 | Not excluded | four periods (20-23, 31-34, 35-37 and 40-41) | ANC | median + SD | p5 - p95 (including median) | Modeled | published algorithm of median | Published algorithm for median and SD |
| Dias^27^ | CPR | 2013-2014 | Crossectional | 596 | 96 | Yes | 14-40 w | ANC | None | p10, p50, p90 en SD | Modeled | calculated from table median | Calculated from table percentile |
| Zohav^28^ | CPR | 2014-2019 | Crossectional | 560 | 560 | Yes | 29-42 w | ANC | None | min, p5 - p75, max, mean + SD | Observed | calculated from table median | Calculated from table percentile |
| Acharya^24^ | CPR and UCR | 2004-2012 | Longitudinal | 284 | 979 | Low risk at inclusion, but later pathology not excluded | from 17-23 w | ANC | percentiles, median, mean +SD | p2.5 - p97.5 | Modeled | published algorithm of median | Published algorithm for percentile |
